# Supplementary material for: Development of a nucleoside-modified mRNA vaccine against clade 2.3.4.4b H5 highly pathogenic avian influenza virus
Source: Nat Commun. 2024 May 23;15:4350. doi: 10.1038/s41467-024-48555-z (PMC11116520; doi:10.1038/s41467-024-48555-z)
Supplement: Supplementary file 1 — Supplementary Information [file 41467_2024_48555_MOESM1_ESM.pdf]

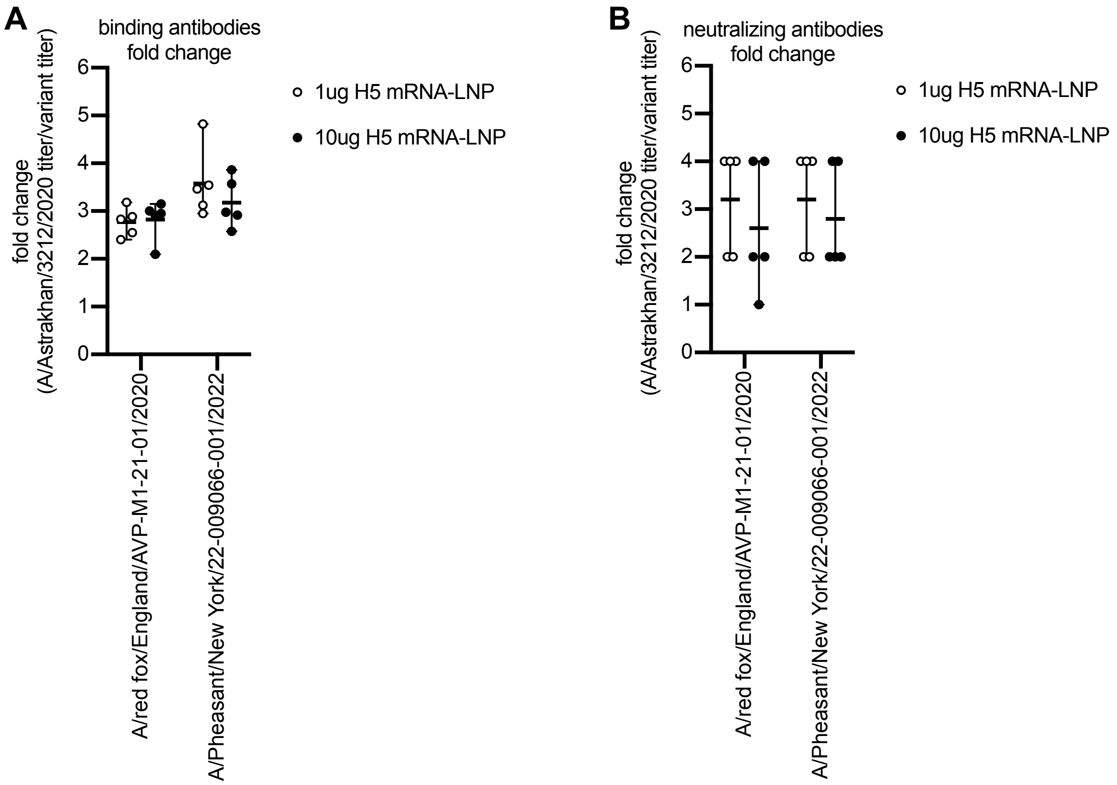

Supplementary Figure 1. Comparison of murine antibody titers against different Clade 2.3.4.4b H5 antigens. Serum samples were collected from mice 28 days after vaccination. ELISA binding and neutralization titers are reported in **Figures 1-2**. Fold change was determined by dividing antibody titers obtained using A/Astrakhan/3212/2020 antigens with titers obtained using either A/red fox/England/AVP-M1-21-01/2020 or A/pheasant/New York/22-009066-001/2022 antigens. Shown are fold change for (A) ELISA binding and (B) neutralization titers. Shown are mean and range. n=5 mice/group

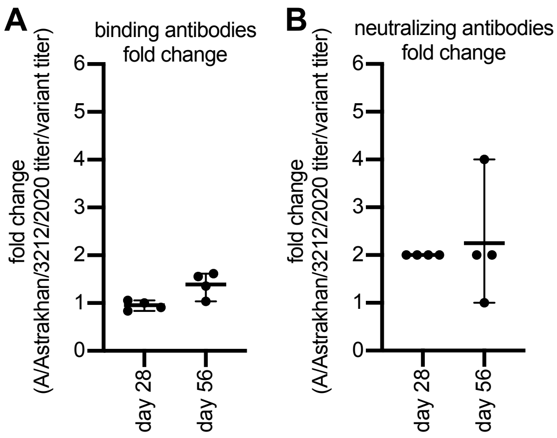

Supplementary Figure 2. Comparison of ferret antibody titers against different Clade 2.3.4.4b H5 antigens. Serum samples were collected from ferrets 28 and 56 days after vaccination. ELISA binding and neutralization titers are reported in **Figure 6**. Fold change was determined by dividing antibody titers obtained using A/Astrakhan/3212/2020 antigens with titers obtained using A/pheasant/New York/22-009066-001/2022 antigens. Shown are fold change for (A) ELISA binding and (B) neutralization titers. Shown are mean and range. n=4 ferrets/group
