# Supplementary material for: Development of a nucleoside-modified mRNA vaccine against clade 2.3.4.4b H5 highly pathogenic avian influenza virus
Source: Nat Commun. 2024 May 23;15:4350. doi: 10.1038/s41467-024-48555-z (PMC11116520; doi:10.1038/s41467-024-48555-z)
Supplement: Supplementary file 3 — Reporting Summary [file 41467_2024_48555_MOESM3_ESM.pdf]

Corresponding author(s): Scott E. Hensley

Last updated by author(s): May 10, 2023

Reporting Summary

Nature Portfolio wishes to improve the reproducibility of the work that we publish. This form provides structure for consistency and transparency in reporting. For further information on Nature Portfolio policies, see our Editorial Policies and the Editorial Policy Checklist.

Statistics

For all statistical analyses, confirm that the following items are present in the figure legend, table legend, main text, or Methods section.

n/a	Confirmed	
☐	☒	The exact sample size (*n*) for each experimental group/condition, given as a discrete number and unit of measurement
☐	☒	A statement on whether measurements were taken from distinct samples or whether the same sample was measured repeatedly
☐	☒	The statistical test(s) used AND whether they are one- or two-sided<br>*Only common tests should be described solely by name; describe more complex techniques in the Methods section.*
☒	☐	A description of all covariates tested
☐	☒	A description of any assumptions or corrections, such as tests of normality and adjustment for multiple comparisons
☐	☒	A full description of the statistical parameters including central tendency (e.g. means) or other basic estimates (e.g. regression coefficient) AND variation (e.g. standard deviation) or associated estimates of uncertainty (e.g. confidence intervals)
☐	☒	For null hypothesis testing, the test statistic (e.g. *F*, *t*, *r*) with confidence intervals, effect sizes, degrees of freedom and *P* value noted<br>*Give P values as exact values whenever suitable.*
☒	☐	For Bayesian analysis, information on the choice of priors and Markov chain Monte Carlo settings
☒	☐	For hierarchical and complex designs, identification of the appropriate level for tests and full reporting of outcomes
☒	☐	Estimates of effect sizes (e.g. Cohen's *d*, Pearson's *r*), indicating how they were calculated

Our web collection on statistics for biologists contains articles on many of the points above.

Software and code

Policy information about availability of computer code

Data collection	No software was used for data collection.
Data analysis	GraphPad Prism version 9.3.0

For manuscripts utilizing custom algorithms or software that are central to the research but not yet described in published literature, software must be made available to editors and reviewers. We strongly encourage code deposition in a community repository (e.g. GitHub). See the Nature Portfolio guidelines for submitting code & software for further information.

Data

Policy information about availability of data

All manuscripts must include a data availability statement. This statement should provide the following information, where applicable:

- Accession codes, unique identifiers, or web links for publicly available datasets
- A description of any restrictions on data availability
- For clinical datasets or third party data, please ensure that the statement adheres to our policy

The data generated in this study are provided in the Source Data file. This paper does not report original code.

Research involving human participants, their data, or biological material

Policy information about studies with human participants or human data. See also policy information about sex, gender (identity/presentation), and sexual orientation and race, ethnicity and racism.

Reporting on sex and gender	Human participants were not used in this study.
Reporting on race, ethnicity, or other socially relevant groupings	Human participants were not used in this study.
Population characteristics	Human participants were not used in this study.
Recruitment	Human participants were not used in this study.
Ethics oversight	Human participants were not used in this study.

Note that full information on the approval of the study protocol must also be provided in the manuscript.

Field-specific reporting

Please select the one below that is the best fit for your research. If you are not sure, read the appropriate sections before making your selection.

[x] Life sciences [ ] Behavioural & social sciences [ ] Ecological, evolutionary & environmental sciences

For a reference copy of the document with all sections, see nature.com/documents/nr-reporting-summary-flat.pdf

Life sciences study design

All studies must disclose on these points even when the disclosure is negative.

Sample size	Sample sizes for murine (n=5) and ferret (n=4) experiments were chosen based on similar previously published studies (Arevalo, C. et al., 2020; Kandeil, A. et al., 2022).
Data exclusions	For data presented in Figure 5, 2 mice with prior exposure to A/California/07/2009 that did not develop binding and neutralizing antibodies to A/California/07/2009 were determined to not have been properly infected and were removed from our analyses.
Replication	All mice experiments were repeated for a total of two independent experiments. For each experiment, mouse serum samples were tested by ELISA and in vitro neutralization assay and reproducibility was confirmed across the independent experiments. Ferret serum samples were tested in 2 independent in vitro neutralization assays. All attempts to repeat experiments were successful.
Randomization	Mice and ferrets were randomly allocated into experimental groups.
Blinding	Blinding was not possible as same person who performed the experiment also collected the data and performed the analysis.

Reporting for specific materials, systems and methods

We require information from authors about some types of materials, experimental systems and methods used in many studies. Here, indicate whether each material, system or method listed is relevant to your study. If you are not sure if a list item applies to your research, read the appropriate section before selecting a response.

Materials & experimental systems

n/a	Involved in the study	
[ ]	[x]	Antibodies
[ ]	[x]	Eukaryotic cell lines
[x]	[ ]	Palaeontology and archaeology
[ ]	[x]	Animals and other organisms
[x]	[ ]	Clinical data
[x]	[ ]	Dual use research of concern
[x]	[ ]	Plants

Methods

n/a	Involved in the study	
[x]	[ ]	ChIP-seq
[x]	[ ]	Flow cytometry
[x]	[ ]	MRI-based neuroimaging

Antibodies

Antibodies used	Horseradish-peroxidase-conjugated goat anti-mouse IgG (Jackson, product number 115-035-003), horseradish-peroxidase-conjugated anti-ferret IgG (Abcam, product number ab112770), horseradish-peroxidase-conjugated anti-human IgG (Jackson, product number 109-036-098), anti-IFNg (Ebioscience; Product number 11-7311-41), anti-Foxp3 (Ebioscience; Product number

	35-5773-82), anti-CXCR5-biotin (Ebioscience, Product number 13-7185-82), Streptavidin-AF647 (Biolegend, Product number 405237), anti-CD90.2 (Biolegend, product number 140324), Ghostdye Red 780 (Tonbo, product number 13-0865-T100), anti-KLRG1 (BC, product number 740279), anti-CD4 (BD, product number 612952), anti-CD8a (BD, product number 752637), anti-CD122 (BD, product number 741537), anti-CD69 (BD, product number612793), anti-CD11a (BD, product number741919), anti-TNFa (Biolegend, product number 506328), anti-PD-1 (Biolegend, product number 135220), anti-CD19 (Biolegend, product number 115541), anti-CD62L (Biolegend, product number104445), anti-CD44 (Biolegend, product number 103059), anti-IL-4 (Biolegend, product number 504104), anti-Bcl-6 (BD, product number 562401), anti-T-bet (Ebioscience, product number 15-5825-82), anti-CD107a (Biolegend, product number 121620), R-Phycoerythrin AffiniPure Goat Anti-Mouse IgG (subclasses 1+2a+2b+3), Fcγ Fragment Specific (Jackson Immunoresearch, product number 115-115-164), Mouse Anti-Human IgG Fc-PE (JDC-10) (SouthernBiotech, product number 9040-09), Mouse anti-influenza A NP clone IC5-1B7 (Produced in house), Horseradish-peroxidase-conjugated rat anti-mouse kappa clone 187.1 (SouthernBiotech, product number 1170-05).
Validation	From the manufacturer's datasheets:<br>For Jackson 115-035-003: Based on immunoelectrophoresis and/or ELISA, the antibody reacts with whole molecule mouse IgG. It also reacts with the light chains of other mouse immunoglobulins. No antibody was detected against non-immunoglobulin serum proteins. The antibody may cross-react with immunoglobulins from other species (Jackson web page).<br>For Abcam ab112770: By immunoelectrophoresis and ELISA the antibody reacts specifically with Ferret IgG and with light chains common to other Ferret immunoglobulins. No antibody was detected against nonimmunoglobulin serum proteins. May cross react with IgG from other species (Abcam web page).<br>For Jackson 109-036-098: Based on immunoelectrophoresis and/or ELISA, the antibody reacts with the Fc portion of human IgG heavy chain but not with the Fab portion of human IgG. No antibody was detected against human IgM or IgA, or against non-immunoglobulin serum proteins. The antibody has been tested by ELISA and/or solid-phase adsorbed to ensure minimal cross-reaction with bovine, horse and mouse serum proteins, but it may cross-react with immunoglobulins from other species (Jackson web page).<br>For Ebioscience 11-7311-41, 35-5773-82, : These Antibodies were verified by Cell treatment to ensure that the antibody binds to the antigen stated (ThermoFisher web page).<br>For Biolegend 140324, 506328, 135220, 115541, 104445, 103059, 504104, 121620: Each lot of these antibodies are quality control tested by immunofluorescent staining with flow cytometric analysis (Biolegend web page).<br>For BD 740279: This antibody specifically binds to KLRG1 (Killer cell Lectin-like Receptor G1), which is the mouse homolog of the rat mast cell function-associated antigen (MAFA), on all mouse strains tested (eg, AKR/J, BALB/c, C3H/HeN, C3H.SW, C57BL/6, DBA/1, SJL, 129/J) (BD Web page).<br>For BD 612952: This antibody specifically binds to the mouse CD4 (L3T4) differentiation antigen (BD Web page).<br>For BD 752637: This antibody specifically recognizes CD8a which is also known as CD8 alpha (CD8α), Ly-2 or Lyt-2 (BD Web page).<br>For BD 741537: This antibody specifically binds to mouse CD122 (BD Web page).<br>For BD 612793: This antibody specifically binds to CD69 (Very Early Activation antigen), an 85 kDa disulfide-linked homodimer of differentially glycosylated subunits (BD web page).<br>For BD 741919: This antibody specifically binds to the 180-kDa αL chain of LFA-1 (CD11a/CD18, αLβ2 integrin), a heterodimeric surface glycoprotein expressed on almost all leukocytes (BD web page).<br>For BD 562401: This antibody specifically binds to Bcl-6 (BD web page).<br>For Ebioscience 13-7185-82: This antibody was verified to be specific for CXCR5 for use in flow cytometry. Publications: PMID 33296685, PMID 28114383, PMID 29249358.<br>For Ebioscience 15-5825-82: This antibody has been verified for use in intracellular staining followed by flow cytometric analysis. Publications: PMID 32572163, PMID 31801070, PMID 31042472.<br>For Jackson 115-115-164: Based on immunoelectrophoresis and/or ELISA, the antibody reacts with mouse IgG subclasses 1, 2a, 2b, and 3; but not with the Fab portion of mouse immunoglobulins. No antibody was detected against mouse IgM or non-immunoglobulin serum proteins. The antibody has been tested by ELISA and/or solid-phase adsorbed to ensure minimal cross-reaction with human, bovine, and rabbit serum proteins, but it may cross-react with immunoglobulins from other species.

Eukaryotic cell lines

Policy information about cell lines and Sex and Gender in Research

Cell line source(s)	Madin–Darby canine kidney (MDCK) cells (ATCC CCL-34) were used to quantify ferret nasal wash viral titers.<br>MDCK-Siat1-TMPRSS2-PB1 cells and 293T-CMV-PB1 cells were obtained from Dr. Jesse Bloom (Fred Hutchinson Cancer Center). These cells were used for generating viruses by reverse genetics or for in vitro neutralization assays.<br>293F suspension cells were derived from primary embryonal human kidney transformed with sheared human adenovirus type 5 DNA.
Authentication	The MDCK and 293T cells described above are routinely used in our laboratory for influenza virus assays and 293F cells are routinely used in our laboratory for protein production and they were not specifically authenticated for this study. Mycoplasma testing occurs regularly.
Mycoplasma contamination	All cell lines tested negative for mycoplasma contamination.
Commonly misidentified lines<br>(See ICLAC register)	None were used in this study.

Animals and other research organisms

Policy information about studies involving animals; ARRIVE guidelines recommended for reporting animal research, and Sex and Gender in Research

Laboratory animals	Female C57BL/6 mice (obtained from Charles River Laboratories) aged 6-8 weeks were used in this study. Influenza-seronegative male ferrets aged four-to-six-month-old (obtained from Triple F Farms, Sayre, PA, USA) were used in this study.
Wild animals	The study did not involve wild animals.
Reporting on sex	We measured vaccine elicited immune responses in female (mice) and male (ferret) animals. Female mice are typically used for research due to easier husbandry. Male ferrets are typically used for research since there can be estrus-related health problems in female ferrets (Ball, R., 2006).
Field-collected samples	The study did not involve samples collected from the field.
Ethics oversight	Mouse studies were approved by the Institutional Animal Care and Use Committees of the Wistar Institute and the University of Pennsylvania. Ferret studies were approved by the St. Jude Children's Research Hospital Institutional Animal Care and Use Committee.

Note that full information on the approval of the study protocol must also be provided in the manuscript.
